# Simultaneous measurements of top surface and its underlying film surfaces in multilayer film structure

**DOI:** 10.1038/s41598-017-11825-6

**Published:** 2017-09-19

**Authors:** Young-Sik Ghim, Hyug-Gyo Rhee, Angela Davies

**Affiliations:** 1 Center for Space Optics, Korea Research Institute of Standards and Science (KRISS), Science Town, Daejeon, 34113 South Korea; 2 Department of Science of Measurement, University of Science and Technology(UST), Science Town, Daejeon, 34113 South Korea; 3 Department of Physics and Optical Science, University of North Carolina at Charlotte(UNCC), 9201 University City Boulevard, Charlotte, North Carolina 28223 USA

## Abstract

With the growth of 3D packaging technology and the development of flexible, transparent electrodes, the use of multilayer thin-films is steadily increasing throughout high-tech industries including semiconductor, flat panel display, and solar photovoltaic industries. Also, this in turn leads to an increase in industrial demands for inspection of internal analysis. However, there still remain many technical limitations to overcome for measurement of the internal structure of the specimen without damage. In this paper, we propose an innovative optical inspection technique for simultaneous measurements of the surface and film thickness corresponding to each layer of multilayer film structures by computing the phase and reflectance over a wide range of wavelengths. For verification of our proposed method, the sample specimen of multilayer films was fabricated via photolithography process, and the surface profile and film thickness of each layer were measured by two different techniques of a stylus profilometer and an ellipsometer, respectively. Comparison results shows that our proposed technique enables simultaneous measurements of the top surface and its underlying film surfaces with high precision, which could not be measured by conventional non-destructive methods.

## Introduction

Multilayer circuits consisted of a stack of transparent conductive thin-film layers have been widely applied as the core and high-tech components throughout entire industries including semiconductor, flat panel display, and LED industries. In the semiconductor industry, the development of a stacked package technology leads to a new paradigm for manufacturing process by providing a volumetric packaging solution for higher integration and performance, and new products with 3D packaging technology meets the needs of more diverse and complex consumers. There are increasing demands for inspection of the internal structure of multilayer films. In the display industry, an organic luminescent electronic display (OLED) with multilayer transparent electrodes has emerged as the new generation display technique to provide superior mechanical flexibility, electrical conductivity, and optical transparency. It requires field inspection solutions in order to improve productivity. In photovoltaic cells and LED lamps, which are attracting attention as clean energy source, the metrology of thin film structure is also required to control the manufacturing process.

The most reliable and intuitive film thickness analysis is the cross-section observation with a scanning electron microscope (SEM). But this technique is time consuming for specimen preparation and damages the specimen itself. Non-destructive methods using ellipsometry^1–3^ and reflectometry^3^ are widely used for thin film characterization in industrial field. These techniques measure the variation of polarization or intensity upon reflection and compare it to a theoretical model. However, they are basically a point by point thickness measurement and not suitable for 3D inspection of the internal structure of complex multilayer films.

Recently, in response to industrial needs, much research has been done on 3D inspection of complex thin-film structures using white-light interferometry^4–23^. These approaches allow simultaneous measurements of top surface and its underlying film surfaces with high precision, but their applications are only limited to measurement of a single layer film structure. To further effort to expand into multilayer films, other research attempt^23^ has been performed by utilizing both the phase and reflectance spectra of sample to measure individual film thickness of each layer in a stack with more improved precision. However, this technique selects only several phase and reflectance spectra with corresponding filters available with narrow bandpass regions and polarization components as phase shifters such as wave plates also inherently introduce the wavelength-dependent errors in the phase shift. The wavelength range available for measurement significantly influences on the measurement results because the wider the wavelength band, the more the reflectance and phase information at every wavelength on the specimen can be obtained. Hence, obtaining a wide range of spectral wavelengths is essential in order to improve measurement accuracy, stability, and precision for the thin-film metrology.

In this paper, in an attempt to overcome the technical limitations of existing conventional methods, we describe a Linnik interferometric configuration based on spectrally-resolved white-light interferometry. This proposed technique enables accurate measurements of the phase and reflectance over a large range of wavelengths and these spectral interference signals are used to achieve volumetric inspection of complex multilayer film structure.

## Results

### System configuration

**Figure 1:**
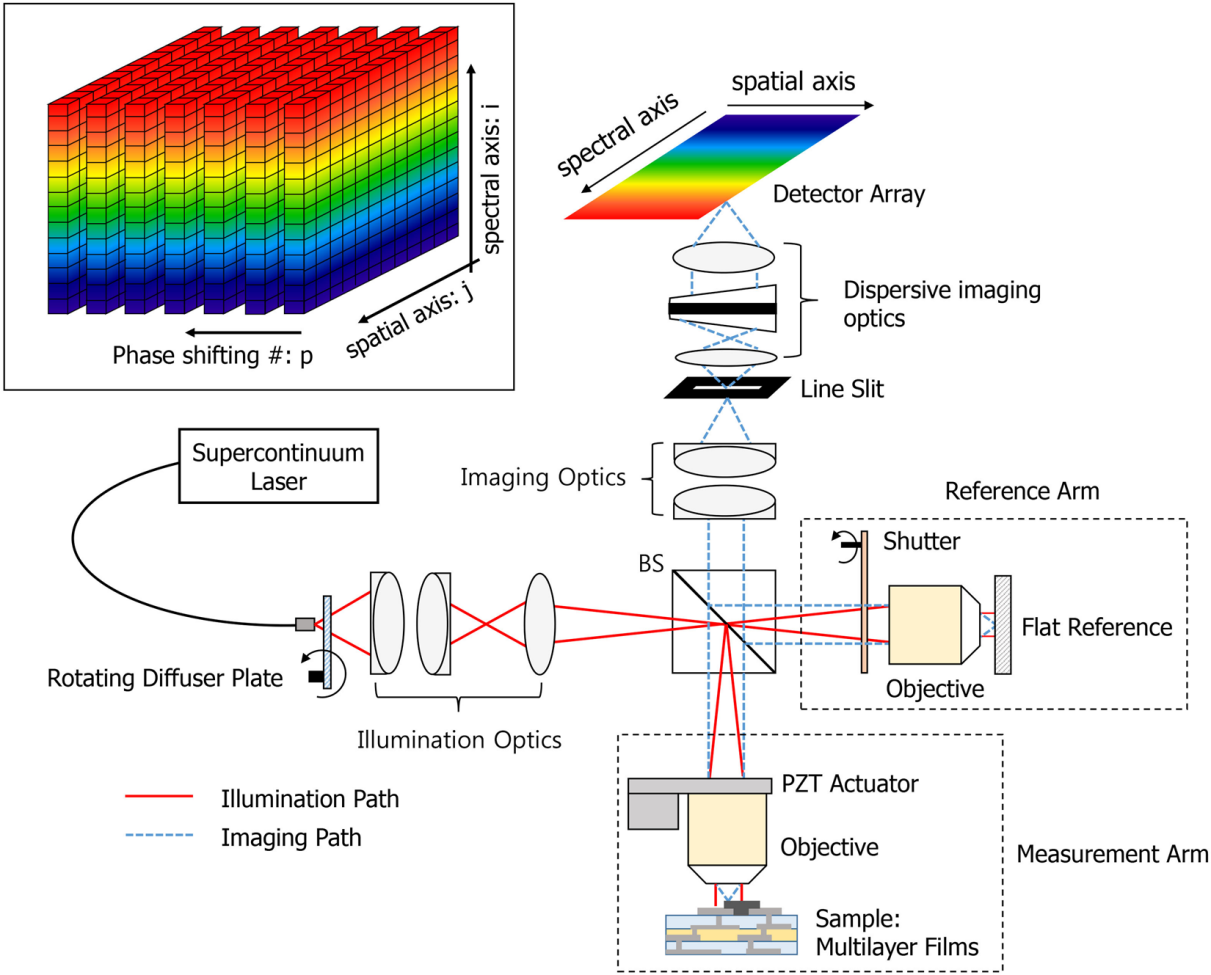
A schematic diagram of our proposed technique for measurement of three-dimensional thickness profile of multilayer film structure.

Figure 1 shows a schematic diagram of our proposed method. A supercontinuum laser with high output power and broad spectrum was used as the light source to obtain the spectral signals as wide as possible. The high-spatial-coherence broadband fiber source generates the speckle noisy pattern, but this random granular pattern can be eliminated using a rotating diffuser plate. Köhler illumination is used to provide a uniformly illuminated field of view of two identical microscope objectives in a Linnik-type configuration. In the reference arm, we generate the reference wave from a flat reference and use a shutter to selectively block (or pass) the reference wave by activating (or deactivating) the shutter. Similarly, the measurement wave is generated from the sample in the measurement arm. According to the operating status of the shutter, we can obtain one of either the reflected beam from the sample or the mutual interference beam between the sample and the reference. The field of view of each microscope objective in two arms (the reference arm and the measurement arm) is delivered into an entry line slit by means of the imaging optics. Then, the line slit and dispersive imaging optics allow the 2D detector array to obtain the spectral information of the pixels on a single spatial line of a sample, of which one axis corresponds to the spatial axis of the selected line and the other corresponds to the spectral axis. In the measurement arm, we use a piezoelectric actuator (PZT) to acquire several sequential phase-shifted spectral images and an iterative least-squares phase-shifting algorithm is used to measure accurately the broadband phase spectra by suppressing serious phase-shift errors. In general, it is necessary to introduce an expected amount of phase shift at equal intervals based on a wavelength of a light source used. However, it is actually difficult to precisely induce the same amounts of phase shifts in accordance with an entire wavelength band of a multi-wavelength light source. Therefore, we adopt an iterative least-squares phase-shifting method which under an assumption that the amount of phase shift is arbitrary regardless of a wavelength of a light source, and calculate the phase information over a wide range of wavelengths only using several phase-shifted spectral interferograms by an iterative operation.

### Phase measurement via iterative least-squares phase-shifting method

The intensity distribution of a 2D detector array in the upper left inset of Fig. 1 can be expressed as follows

1 $$\begin{array}{c}{I}_{ijp}={{I}^{ref}}_{ij}+{{I}^{mea}}_{ij}+2\sqrt{{{I}^{ref}}_{ij}{{I}^{mea}}_{ij}}\,\cos ({{\rm{\Phi }}}_{ij}-{\delta }_{p})\\ ={D}_{ij}+{V}_{ij}\,\cos ({{\rm{\Phi }}}_{ij}-{\delta }_{p}),\end{array}$$

where, *D*_*ij*_ and *V*_*ij*_ represent $({I}_{ij}^{ref}+{I}_{ij}^{mea})$ and ${V}_{ij}=2\sqrt{{I}_{ij}^{ref}{I}_{ij}^{mea}}$, the subscript *i*, *j*, *p* denote the *i*’th pixel number in the spectral axis of a 2D detector array, the *j*’th pixel number in the spatial axis of a 2D detector array, the *p*’th phase shift, respectively. *I* ^*ref*^ and *I* ^*mea*^ are the reference and measurement intensities, Φ is the phase of the test sample to be measured, *δ* is the amount of phase shift controlled by a PZT actuator. In order to measure the phase Φ over a wide range of wavelengths by removing wavelength-dependent phase-shift errors, we used an iterative least-squares phase-shifting algorithm as follows^24–27^. When the first phase shift *δ*_1_ is zero, equation (1) becomes

2 $${I}_{ij1}={D}_{ij}+{V}_{ij}\,\cos \,{{\rm{\Phi }}}_{ij},$$

To eliminate *D*_*ij*_ in the above equations, we can define the intensity difference function *η*_*ijp*_ as

3 $${\eta }_{ijp}={I}_{ijp}-{I}_{ij1}={C}_{ij}(\cos \,{\delta }_{p}-1)+{S}_{ij}\,\sin \,{\delta }_{p},$$

where, *C*_*ij*_ = *V*_*ij*_cosΦ_*ij*_ and *S*_*ij*_ = *V*_*ij*_sinΦ_*ij*_. When it is assuming that the measured value of *η*_*ijp*_ is *η*′_*ijp*_, the error function of *E*_*ij*_ can be defined by the least squares method as follows

4 $${E}_{ij}={\sum _{p=1}^{m}({\eta }_{ijp}-{\eta }_{ijp}^{^{\prime} })}^{2}={\sum _{p=1}^{m}({C}_{ij}(\cos {\delta }_{p}-1)+{S}_{ij}\sin {\delta }_{p}-{\eta }_{ijp}^{^{\prime} })}^{2},$$

in which, *m* is the number of phase-shifts taken. In the above equation, there are only two unknown values of *C*_*ij*_ and *S*_*ij*_, which can be found by minimizing the error *E*_*ij*_ with the following condition

5 $$\frac{\partial {E}_{ij}}{\partial {C}_{ij}}=\frac{\partial {E}_{ij}}{\partial {S}_{ij}}=0,$$

After transforming equation (5) into a matrix form, *C*_*ij*_ and *S*_*ij*_ are mathematically calculated as

6 $$\begin{array}{c}[\begin{array}{c}{C}_{ij}\\ {S}_{ij}\end{array}]\,=\\ {[\begin{array}{cc}\sum _{p=1}^{m}{(\cos {\delta }_{p}-1)}^{2} & \sum _{p=1}^{m}\sin {\delta }_{p}{(\cos {\delta }_{p}-1)}^{2}\\ \sum _{p=1}^{m}\sin {\delta }_{p}{(\cos {\delta }_{p}-1)}^{2} & \sum _{p=1}^{m}{(\sin {\delta }_{p})}^{2}\end{array}]}^{-1}[\begin{array}{c}\sum _{p=1}^{m}\eta {\text{'}}_{ijp}(\cos \,{\delta }_{p}-1)\\ \sum _{p=1}^{m}\eta {\text{'}}_{ijp}\,\cos \,{\delta }_{p}\end{array}],\end{array}$$

And the total phase is directly calculated using *C*_*ij*_ and *S*_*ij*_

7 $${{\rm{\Phi }}}_{ij}={\tan }^{-1}\frac{{S}_{ij}}{{C}_{ij}},$$

As described above, Φ_*ij*_ can be obtained without any constraint on the phase shift if the amount of phase shift is known. Hence, it is very critical to know the true value of phase shift *δ*_*p*_ for precise phase measurement. In order to obtain the exact phase shift, another error function *E*_*p*_ can be defined by the least square method as represented in equation (8)

8 $${E}_{p}={\sum _{j=1}^{l}({\eta }_{ijp}-{\eta }_{ijp}^{^{\prime} })}^{2}={\sum _{j=1}^{l}({C}_{ij}(\cos {\delta }_{p}-1)+{S}_{ij}\sin {\delta }_{p}-{\eta }_{ijp}^{^{\prime} })}^{2},$$

in which, *l* is the number of pixels in the spatial axis. Using the similar procedures as described above, the true value of *δ*_*p*_ is a problem for calculating the optimal phase shift, which minimizes the error *E*_*p*_ with the following condition

9 $$\frac{\partial {E}_{p}}{\partial \,\cos \,{\delta }_{p}}=\frac{\partial {E}_{p}}{\partial \,\sin \,{\delta }_{p}}=0,$$

Finally, we can derive the matrix equation as

10 $$\begin{array}{c}[\begin{array}{c}\cos \,{\delta }_{p}\\ \sin \,{\delta }_{p}\end{array}]\,=\\ {{V}_{ij}}^{3}{[\begin{array}{cc}\sum _{j=1}^{n}{(\cos {{\rm{\Phi }}}_{ij})}^{2} & \sum _{j=1}^{n}\cos {{\rm{\Phi }}}_{ij}\sin {{\rm{\Phi }}}_{ij}\\ \sum _{j=1}^{n}\cos {{\rm{\Phi }}}_{ij}\sin {{\rm{\Phi }}}_{ij} & \sum _{j=1}^{n}{(\sin {{\rm{\Phi }}}_{ij})}^{2}\end{array}]}^{-1}[\begin{array}{c}\sum _{j=1}^{n}\eta {\text{'}}_{ijp}\,\cos \,{{\rm{\Phi }}}_{ij}+\sum _{j=1}^{n}{(\cos {{\rm{\Phi }}}_{ij})}^{2}\\ \sum _{j=1}^{n}\eta {\text{'}}_{ijp}\,\sin \,{{\rm{\Phi }}}_{ij}+\sum _{j=1}^{n}\cos \,{{\rm{\Phi }}}_{ij}\,\sin \,{{\rm{\Phi }}}_{ij}\end{array}],\end{array}$$

And it leads the solution of phase shift

11 $${\delta }_{p}={\tan }^{-1}\frac{\sin \,{\delta }_{p}}{\cos \,{\delta }_{p}},$$

As described above, equation (7) is a formula to calculate Φ_*ij*_ under the assumption that *δ*_*p*_ is known, and equation (11) is a formula to calculate *δ*_*p*_ under the assumption that Φ_*ij*_ is known. Hence, this is an iterative process by repeating a series of sequential operations until it reaches to the desired value of Φ_*ij*_ and *δ*_*p*_ at all measuring points. Throughout these iterative operations, the phase corresponding to each wavelength of the used pixels on a spectral axis can be measured precisely.

### Film thickness measurements

In case of multilayer films, the total phase variations Φ(*h*,*d*_1_…*d*_*n*_;*k*) according to the wavenumber *k* includes the geometrical parameters related to the top surface height *h* and each layer’s film thickness *d*_1_…*d*_*n*_ as

12 $$\begin{array}{rcl}{\rm{\Phi }}(h,{d}_{1}\cdots {d}_{n};k) & = & 2kh+\psi ({d}_{1}\cdots {d}_{n};k)\\  & = & 2kh+{\psi }_{l}({d}_{1}\cdots {d}_{n};k)+{\psi }_{nl}({d}_{1}\cdots {d}_{n};k)\\  & = & {{\rm{\Phi }}}_{l}(h,{d}_{1}\cdots {d}_{n};k)+{{\rm{\Phi }}}_{nl}({d}_{1}\cdots {d}_{n};k),\end{array}$$

where, *ψ* represents a phase term due to the multiple reflections within the multilayer films, which can be decomposed into a linear phase term *ψ*_*l*_ and a non-linear phase term *ψ*_*nl*_. This non-linear phase term *ψ*_*nl*_ is theoretically equal to the residual term Φ_*nl*_ subtracted the linear part from the total phase term. So, the non-linear part of the total phase Φ_*nl*_(*d*_1_…*d*_*n*_;*k*) is only made as a function of each layer’s film thickness^21^. After obtaining the total phase using the iterative least square phase-shifting method, the shutter in the reference arm is activated to block the reference wave and only the reflected beam from the sample is received in the detector array. From the measurement wave from the sample, we need to obtain additional information such as the spectral reflectance of the sample for more precise measurements of film thickness. However, this measurement wave does not accurately reflect the spectral reflectance because its value includes various properties of all the optical components used in our set-up. In practice, it is not easy to take into account all the optical properties of components used. Instead, we can calibrate these effects by doing the measurement of relative reflectance^3^. We measure the ratio of the intensity of a light reflected from the sample specimen *I* ^*sam*^ to the intensity of the same light beam reflected from the standard specimen *I* ^*std*^. Here we selected a bare crystalline silicon wafer as the standard specimen, because its spectral reflectance is well known in the literature. So, the spectral reflectance of the sample ℜ^*sam*^ can be calculated using equation (13) by measuring relative reflectance of two measurement waves reflected from the sample under test and the standard specimen, respectively. As same to the non-linear phase component Φ_*nl*_(*d*_1_…*d*_*n*_;*k*), the spectral reflectance of the sample is also a function of each layer’s film thickness as

13 $${\Re }^{sam}({d}_{1}\cdots {d}_{n};k)=\frac{{I}^{sam}({d}_{1}\cdots {d}_{n};k)}{{I}^{std}(k)}{\Re }^{ref}(k),$$

Here the spectral reflectance of the standard specimen ℜ^*ref*^ is directly calculated by Fresnel’s equation for reflection using the optical constants of a bare crystalline silicon wafer: *n*(*k*) the refractive index and *κ*(*k*) the extinction coefficient varying with wavenumber.

14 $${\Re }^{ref}(k)=\frac{{(n(k)-1)}^{2}+\kappa {(k)}^{2}}{{(n(k)+1)}^{2}+\kappa {(k)}^{2}},$$

By combining the nonlinear part of the total phase and the absolute reflectance of the test specimen together, we can define a merit function as a function of each layer’s film thickness of the multilayer films as follows

15 $$\begin{array}{c}\xi ({d}_{1}\cdots {d}_{n};{k}_{i})=\\ \sum _{i=1}^{w}\{\alpha {|{{{\rm{\Phi }}}_{nl}}^{E}({k}_{i})-{{\psi }_{nl}}^{T}({d}_{1}\cdots {d}_{n};{k}_{i})|}^{2}+\chi {|{\Re }^{E}({k}_{i})-{\Re }^{T}({d}_{1}\cdots {d}_{n};{k}_{i})|}^{2}\},\end{array}$$

where Φ_*nl*_^*E*^ and ℜ^*E*^ are the measured non-linear phase and spectral reflectance, ψ_*nl*_^T^ and ℜ^*T*^ are the analytical models of the non-linear phase and spectral reflectance derived from the Fresnel’s equations for the multilayer^1,21,23^. The superscript ‘E’ and ‘T’ represents ‘experiment’ and ‘theory’, respectively. *α* and *χ* are the weighting factors to adjust the influence ratio of the nonlinear phase value to the absolute reflectance in the error function. That is, since convergence of the metric function and accuracy of the film thickness vary depending on the weights of *α* and *χ*, these two factors need to be tuned in accordance with the experimental situation. The analytical phase and reflection can be calculated from the optical model as

16 $$\begin{array}{rcl}{\rm{{\rm Z}}} & = & {\Re }^{T}\exp (-j{\psi }^{T})\\ {\Re }^{T} & = & |{\rm{{\rm Z}}}|\\ {\psi }_{nl}^{T} & = & \mathrm{non}-\mathrm{linear}\,{\rm{phase}}\,o{\rm{f}}\,{\psi }^{T},\end{array}$$

In case of three-layer films deposited on a substrate, the reflectivity model Z can be expressed as

17 $$\begin{array}{c}{\rm{{\rm Z}}}=\\ \frac{{r}_{01}{e}^{-j({\beta }_{1}+{\beta }_{2}+{\beta }_{3})}+{r}_{12}{e}^{j({\beta }_{1}-{\beta }_{2}-{\beta }_{3})}+{r}_{23}{e}^{j({\beta }_{1}+{\beta }_{2}-{\beta }_{3})}+{r}_{34}{e}^{j({\beta }_{1}+{\beta }_{2}+{\beta }_{3})}+{r}_{01}{r}_{12}{r}_{23}{e}^{-j({\beta }_{1}-{\beta }_{2}+{\beta }_{3})}+{r}_{01}{r}_{12}{r}_{34}{e}^{-j({\beta }_{1}-{\beta }_{2}-{\beta }_{3})}+{r}_{01}{r}_{23}{r}_{34}{e}^{-j({\beta }_{1}+{\beta }_{2}-{\beta }_{3})}+{r}_{12}{r}_{23}{r}_{34}{e}^{j({\beta }_{1}-{\beta }_{2}+{\beta }_{3})}}{{e}^{j({\beta }_{1}+{\beta }_{2}+{\beta }_{3})}+{r}_{01}{r}_{12}{e}^{-j({\beta }_{1}-{\beta }_{2}-{\beta }_{3})}+{r}_{01}{r}_{23}{e}^{-j({\beta }_{1}+{\beta }_{2}-{\beta }_{3})}+{r}_{12}{r}_{23}{e}^{j({\beta }_{1}-{\beta }_{2}+{\beta }_{3})}+{r}_{01}{r}_{34}{e}^{-j({\beta }_{1}+{\beta }_{2}+{\beta }_{3})}+{r}_{12}{r}_{34}{e}^{j({\beta }_{1}-{\beta }_{2}-{\beta }_{3})}+{r}_{23}{r}_{34}{e}^{j({\beta }_{1}+{\beta }_{2}-{\beta }_{3})}+{r}_{01}{r}_{12}{r}_{23}{r}_{34}{e}^{-j({\beta }_{1}-{\beta }_{2}+{\beta }_{3})}},\end{array}$$

where *r*_*ij*_ is the Fresnel reflection coefficients of the *ij* film layer interface, and *β*_*i*_ is the phase shift when passing through the *i* ^th^ film layer with thickness *d*_*i*_ and complex refractive index *N*_*i*_ expressed as (the subscripts 0, 1, 2, 3 and 4 identify the substrate layer, 1^st^ film layer, 2^nd^ film layer, 3^rd^ film layer, and air layer, respectively)

18 $$\begin{array}{c}{r}_{ij}=\frac{{N}_{i}-{N}_{j}}{{N}_{i}+{N}_{j}}\\ {\beta }_{i}=2k{N}_{i}{d}_{i},\end{array}$$

The complex refractive index of each film layer can be determined by adjusting mathematical model parameters describing the optical constants of materials until the calculated reflectance and phase spectra matches the measured reflectance and phase spectra. However, since optimization of film thickness and refractive index at the same time deteriorates the measurement accuracy, we performed optimization of film thickness only for the sample specimen with known refractive indices. The global optimization method based on genetic algorithm was used to search the unknown variables of *d*_1_…*d*_*n*_ by minimizing the total sum over the entire wave-number range in equation (15).

### Surface profile measurements

Based on the film thickness information obtained by the procedure described above, another merit function can be defined similarly so as to measure the top surface profile *h*

19 $${\rm{\Gamma }}(h)={\sum _{i=1}^{w}|{{\rm{\Phi }}}^{E}({k}_{i})-{\psi }^{T}({d}_{1}\cdots {d}_{n},{k}_{i})-2{k}_{i}h|}^{2},$$

in which, Φ^*E*^ and ψ^T^ indicate the measured total phase term and the analytical phase term computed by substituting the predetermined film thickness value into Fresnel’s equation for a multilayer film. Therefore, we can reconstruct volumetric film thickness profile using equations (15) and (19).

### Fabrication of multilayer film structure

**Figure 2:**
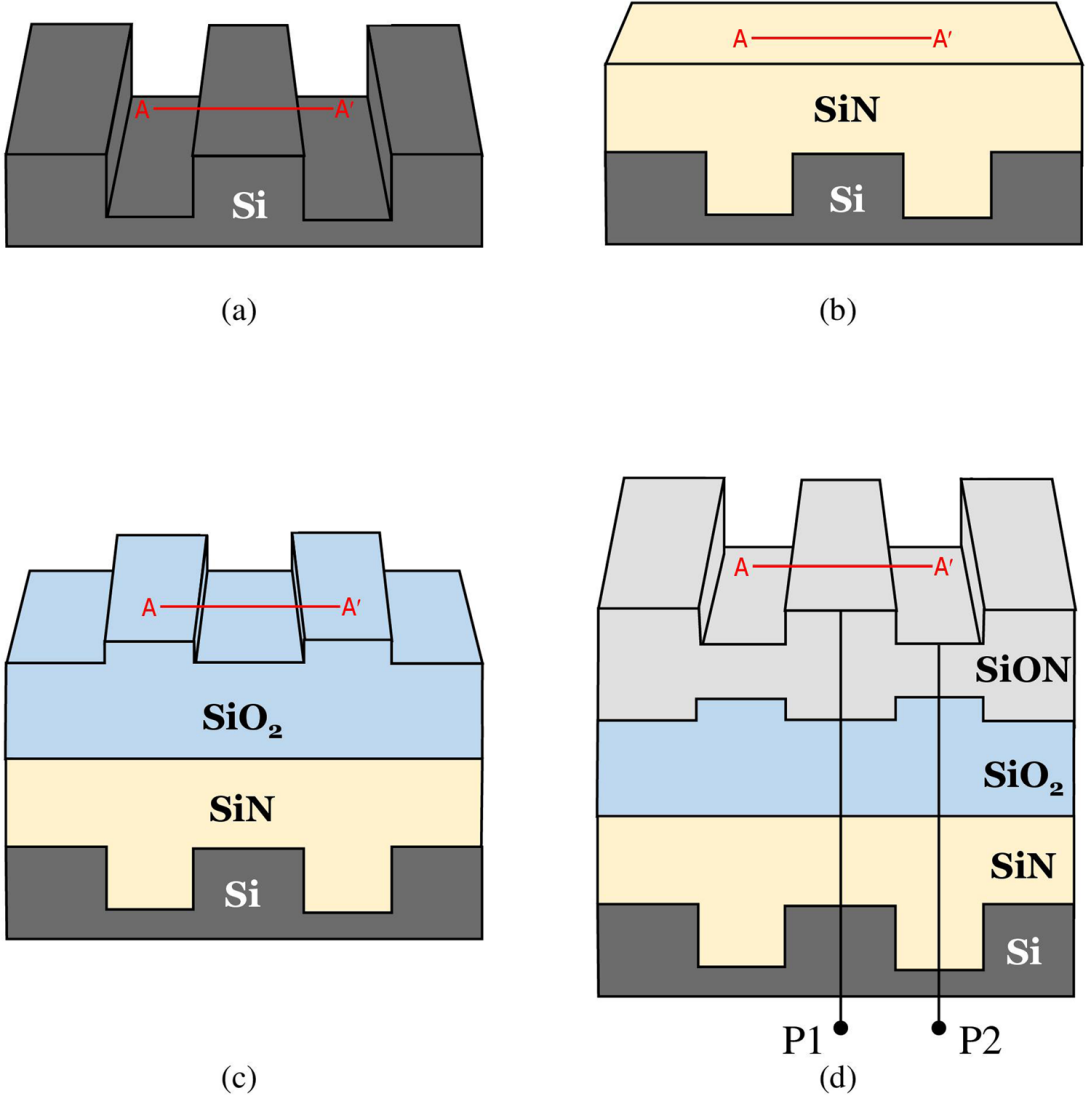
Schematic illustrations of the fabrication process of multilayer film structure: (**a**) pattering of a bare Si wafer, (**b**) SiN film deposition on a patterned Si wafer and planarization by a subsequent CMP, (**c**) SiO_2_ film deposition on a SiN layer and subsequent patterning of a SiO_2_ layer, and (**d**) SiON film deposition on a SiO_2_ layer and subsequent patterning of a SiON layer. For comparison of our measurement results with commercial instruments, the surface profile of each layer at A-A′ line was measured by a stylus profiler and the film thickness of each layer at P1 and P2 positions was also measured by an ellipsometer.

For verification of our proposed technique, we manufactured a three-layer film structure via a series of sequential photolithography process. First, a step pattern of about 500 nm was formed on the bare silicon substrate through the etching process (Fig. 2(a)). Then, a SiN film was deposited as 1^st^ layer on the Si patterned structure about 1.6 μm and the upper surface was planarized by the CMP (chemical mechanical polishing) process (Fig. 2(b)). After that, a SiO_2_ film layer of 1.5 μm was deposited as 2^nd^ layer and the etching process was performed in the opposite step pattern to the first etching around 300 nm (Fig. 2 (c)). Finally, a SiON film with a thickness of 1.3 μm was coated on the SiO_2_ layer as 3^rd^ layer, and then an etching process was performed so as to generate the same step pattern to the first etching about 600 nm (Fig. 2(d)). For reference, the surface profile of line A-A′ of each layer was measured at each process step from Fig. 2(a) to (d) using a stylus profiler, and the film thickness at P1 and P2 positions was also measured with an ellipsometer. These measurement results will be used to verify the performance of our proposed technique.

### Experimental results

Table 1 shows details regarding the apparatus used for the measurements. We used a supercontinuum white light source with a wavelength distribution over an almost entire band from a visible to an infrared ray region. However, since a responsible band of the detector is restrictive, a limit of a wavelength band (visible wavelength range from 380 nm to 800 nm) is actually determined by the detector. In our system, the whole measurement process can be divided into two consecutive steps. The first is to measure the spectral reflectance of the sample. By blocking the reference wave in the reference arm by turning the shutter on, we measure the spectral reflectance of the sample specimen ${\Re }^{sam}$ using equation (13). Secondly by turning the shutter off, the interference beam between the measurement wave and the reference wave is obtained. To determine the phase of this interference beam over a broad wavelength range, five spectral interferograms as the minimum number of frames in the iterative least-squares phase-shifting algorithm were generated with the phase shifts of *δ*_1_ = 0, *δ*_2_ = 60 nm, *δ*_3_ = 120 nm, *δ*_4_ = 180 nm, *δ*_5_ = 240 nm using a closed-loop piezo-equipped nano-positioning actuator. These amount of phase shifts are used as initial guesses of *δ*_*p*_^*0*^ for all wavelengths used.

**Table 1:** Specifications of apparatus of our proposed method.

Microscope Objectives	2.5 × with 0.07 N.A.
Spectral range of detector	380 – 800 nm
Spectral resolution	2 nm
Length of the scene line imaged at a time	4.736 mm
Lateral resolution	2.96 μm

**Figure 3:**
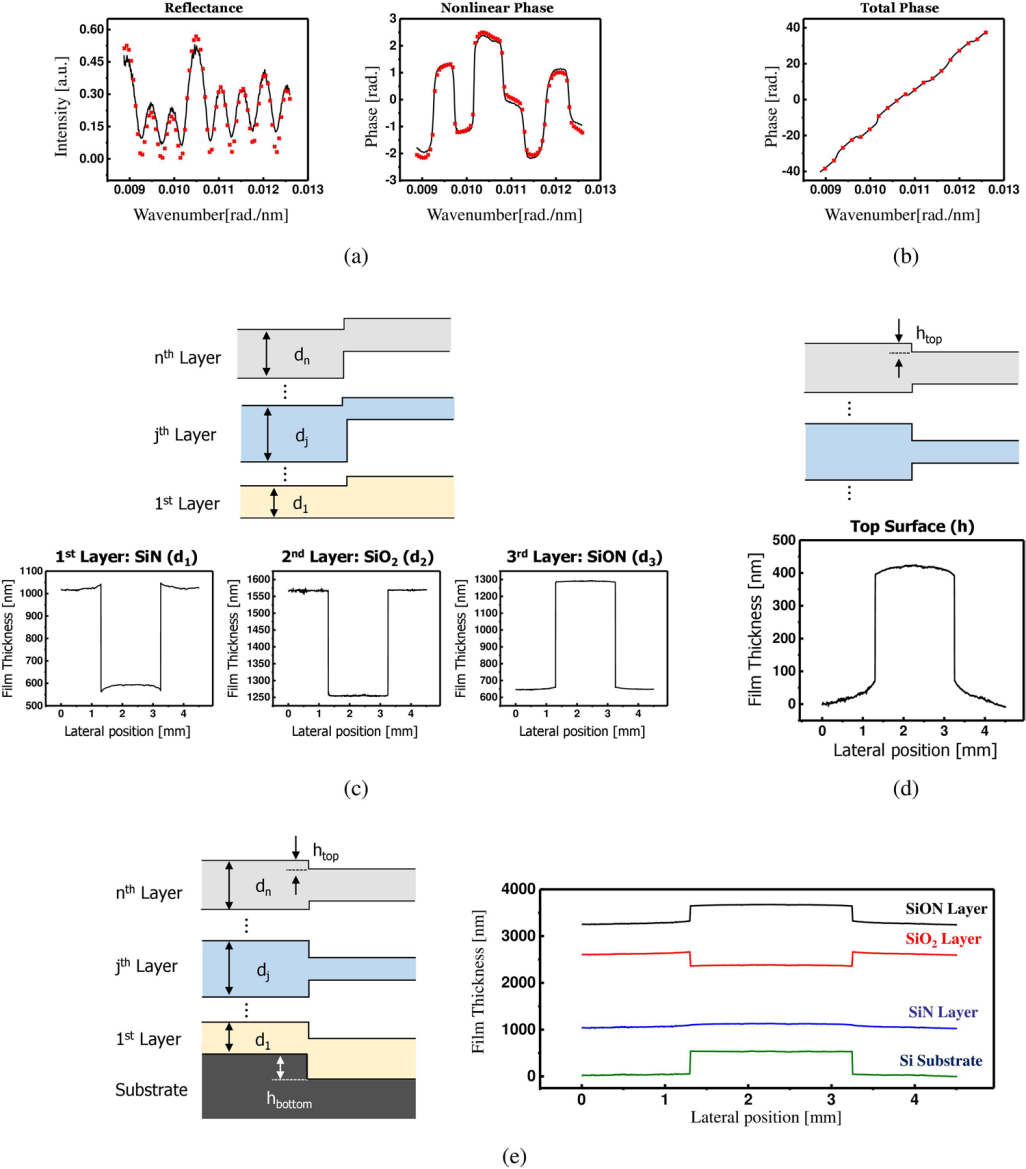
A series of signal procedure of our proposed method: (**a**) comparison results of the absolute reflectance and the non-linear phase component of the measurement (black solid line) and the analytical model (red crosses), respectively. Equation (15) is used for film thickness measurement, (**b**) comparison result of the measured (black solid line) and analytical (red crosses) total phase distributions. Equation (19) is used for surface profile measurement, (**c**) film thickness measurement results of each layer of the specimen of one line via (**a**) procedure, (**d**) surface profile measurement result of the top layer of the specimen of one line via (**b**) procedure, and (**e**) reconstructed film thickness profile of the specimen of one line using measurement results of (**c**) and (**d**).

**Figure 4:**
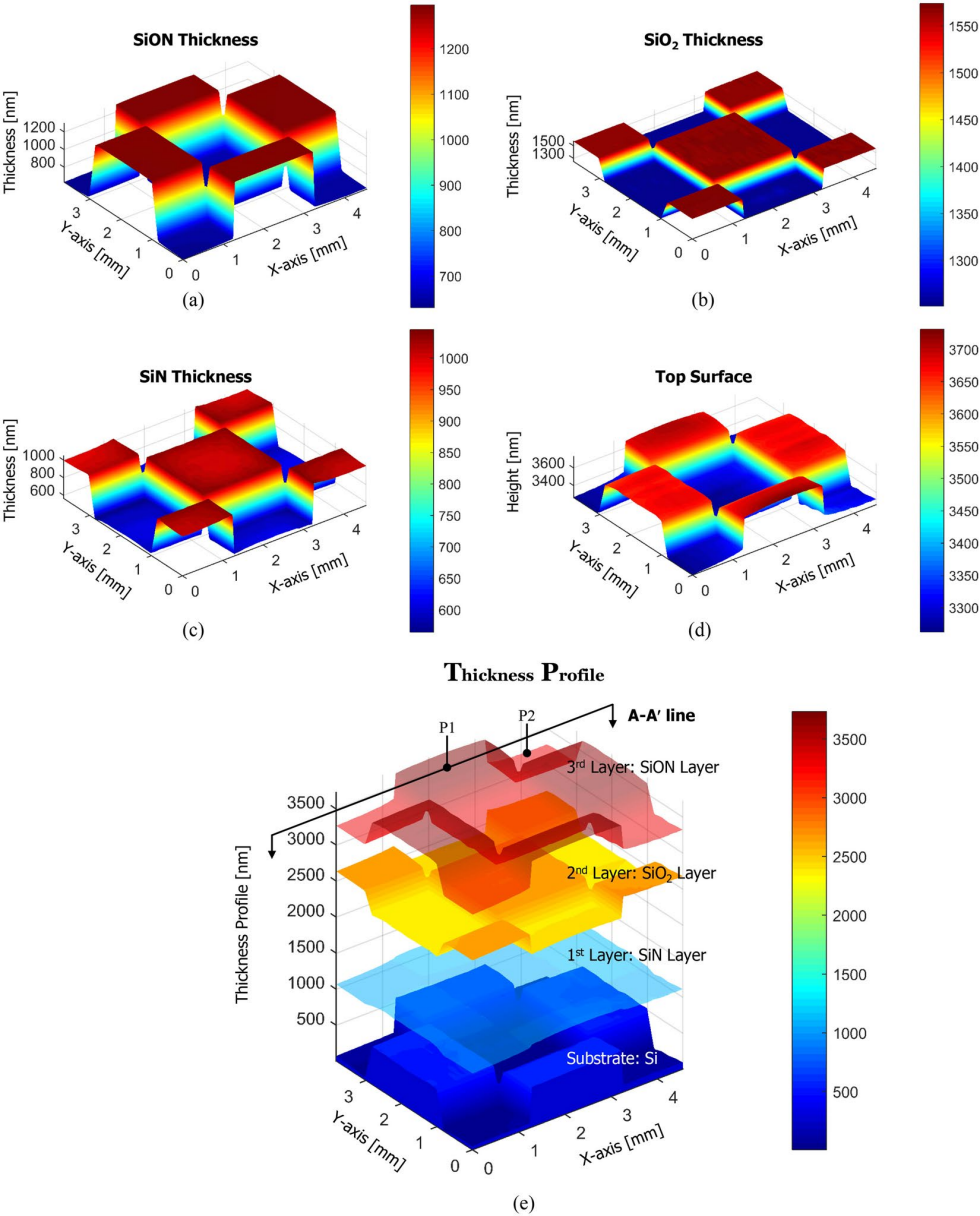
Three-dimensional measurement results by scanning laterally over the specimen line by line using a motorized micro-positioning stage: (**a**) three-dimensional thickness of a SiON film layer (3^rd^ Layer), (**b**) three-dimensional thickness of a SiO_2_ film layer (2^nd^ Layer), (**c**) three-dimensional thickness of a SiN film layer (1^st^ Layer), (**d**) three-dimensional top surface profile (SiON Layer) of the specimen, and (**e**) reconstructed volumetric film thickness profile using measurement results of (**a**) to (**d**).

As iterations proceed, the computed phase values corresponding to each wavelength converge to their true values. Finally, we measured the phase spectra of the sample, which can be divided into the linear and nonlinear phase components as described in equation (12). As shown in Fig. 3(a), the spectral reflectance and nonlinear phase part are used to determine the film thickness of each layer by minimizing the merit function of equation (15) using the global optimization process. Here, the weight factors of *α* and *χ* are adjusted so as to equally contribute the phase and reflectance values on the merit function. As a result, the film thicknesses *d*_1_, *d*_2_, and *d*_3_ of each layer of the sample were obtained. In a similar way, as shown in Fig. 3(b), the true value of the top surface height *h* was also measured by minimizing the error function of equation (19). Through the measurement procedures described above, we separately measured the film thickness and surface height of each layer of a single spatial line of the sample as shown in Fig. 3(c) through (e). For a complete 3D thickness profile measurement, the specimen is scanned in the lateral direction with a sequential step of 100 μm using a motorized micro-positioning stage. Figure 4 presents three-dimensional thickness profile of complex three-layer film structure, which could not be analyzed by conventional non-destructive testing.

### Comparison of measurement results

**Figure 5:**
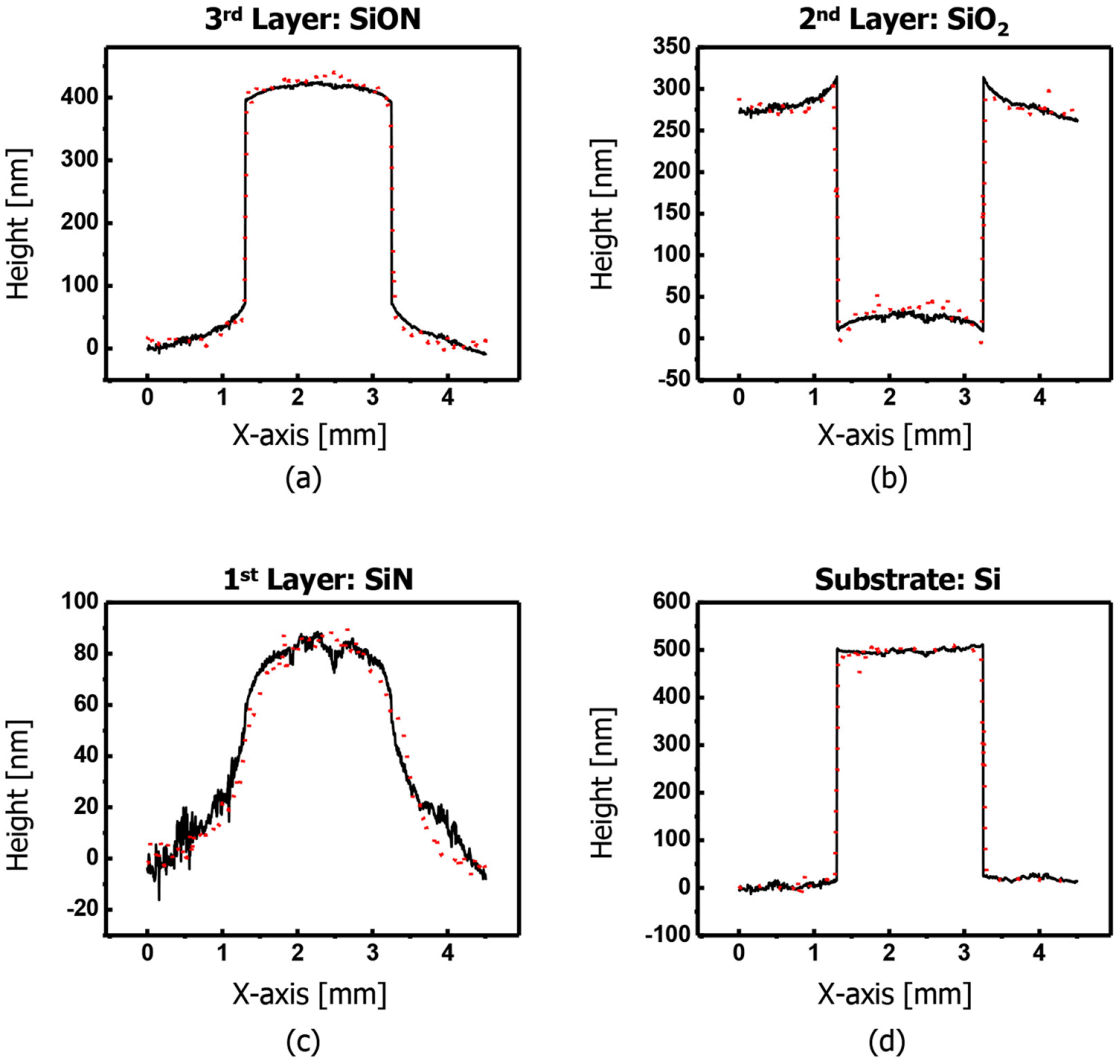
Comparisons of our measurement results (black-solid line) with a Dektak 8 (Veeco) stylus profilometer (red dots) when measuring an A-A′ line profile of the specimen: (**a**) surface profile of a 3^rd^ SiON layer, (**b**) surface profile of a 2^nd^ SiO_2_ layer, (**c**) surface profile of a 1^st^ SiN layer, and (**d**) surface profile of a silicon substrate.

We verified the measurement accuracy of our proposed method through comparisons with two well-known measurement techniques, ellipsometer and stylus profilometer. Table 2 shows the comparison results of the film thickness of each layer with a KLA-Tencor SpectraFx 100 ellipsometer at P1 and P2 positions of the sample. The discrepancy between the results of the two measurement methods is less than ~18 nm. Figure 5 shows another comparison of our measurement results with a Veeco Dektak 8 stylus profilometer when measuring an A-A′ line surface profile of each layer of the sample. For ease of comparison, the minimum values of each line profile measured by two different methods were adjusted close to zero, and these two measured surface profiles are well matched to each other. The differences between two measurement results mainly occurs due to the inconsistency of measurement points and systematic errors. The systematic errors can be corrected by measuring a calibration specimen and applying the measured correction value so as to compensate for a system bias.

**Table 2:** Comparison results with a commercial instrument of ellipsometer.

Sampling Position		KLA-Tencor SpectralFx 100 Ellipsometer	Our Proposed Technique (mean ± σ)
SiN Layer (1^st^ Layer)	P1	581.7 nm	594.3 ± 0.25 nm
	P2	1004.3 nm	1011.5 ± 1.55 nm
SiO_2_ Layer (2^nd^ Layer)	P1	1246.9 nm	1255.2 ± 0.75 nm
	P2	1563.9 nm	1568.0 ± 2.52 nm
SiON Layer (3^rd^ Layer)	P1	1312.1 nm	1294.1 ± 2.06 nm
	P2	641.5 nm	642.6 ± 0.27 nm

## Discussion

To verify our proposed technique, a three-layer complex multilayer film was fabricated as the sample specimen. However, such a complex multilayer film structure is very challenging to measure with conventional measuring technique. So, the surface profile and film thickness of the sample were separately measured by using conventional techniques: a stylus profilometer and an ellipsometer. A stylus profilometer drag a tip probe across the measurement surface, so that it damages the sample as well as it does not provide a surface profile for the underlying structure of the complex multilayer films. So, the surface profile of each layer was measured at each process step before the next film layer deposited. An ellipsometer measures the film thickness by detecting a change in polarization as light reflects from the sample. It is basically a point-by-point thickness measurement and practically not available to measure 3D surface profiling for complex multilayer film structure. For these reasons, it is difficult to directly measure the internal structure of the sample using existing non-destructive methods, so an approximate individual comparison for each film layer’s surface profiling and thickness was performed. Indirect comparison results confirmed that our method successfully enables measurement of three-dimensional thickness profile of multilayer film structure while maintaining high precision same as existing methods. The sample specimen was repeatedly measured at least ten times and the standard deviation for each layer’s film thickness was 2.52 nm or less. The maximum length of a single line that can be measured at a time and dynamic range in the vertical direction are 4.736 mm and up to 100 μm when a microscope objective of 2.5× magnification with 0.07 N.A. is used. These system parameters strongly depend on the specifications of the objective lens used. The calculation time taken for simultaneous measurement of surface profile and film thickness at a single point is worked out approximately 1 second when executing scripts written in MATLAB using a standard PC equipped with an INTEL core i7-4790 3.6 Hz Processor. One of the challenges to be solved for in-line measurement is to reduce dramatically the calculation time. In a simple way, we can significantly improve the computational speed by translating MATLAB code to C++ code. The development of high performance computers and efficient computing algorithms such as parallel computation is also expected as possible solutions sooner or later.

## Conclusion

We proposed a new approach that goes one step further than conventional methods, which provides simultaneous measurement of the film surface and thickness by computing the absolute reflectance and phase over a wide range of wavelengths without any damage to the sample. We used the iterative least-squares phase-shifting algorithm for precise measurement of the broadband phase spectra by suppressing critical phase shift errors, and it provides a better solution for multilayer film metrology. In the near future, it is anticipated that our proposed technique will be widespread as a more general metrological tool for 3D inspection of multilayer film structure in thin-film metrology.
